# Is the Relocation of Polluting Industries Prompted by FDI Flow and Stock, Globalisation, Corruption and Regulation?

**DOI:** 10.3390/ijerph18041981

**Published:** 2021-02-18

**Authors:** Patrícia Hipólito Leal, Rafaela Vital Caetano, António Cardoso Marques

**Affiliations:** 1 Management and Economics Department, University of Beira Interior, 6201-001 Covilhã, Portugal; patricia.leal@ubi.pt (P.H.L.); rafaela.caetano@ubi.pt (R.V.C.); 2 Management and Economics Department NECE-UBI—Research Unit in Business Sciences, University of Beira Interior, Rua Marquês d’Ávila e Bolama, 6201-001 Covilhã, Portugal

**Keywords:** inward and outward Foreign Direct Investment flow and stock, globalisation *de jure* and *de facto*, index for sustainable economic welfare, corruption, regulation quality, environmental degradation

## Abstract

Can globalisation and foreign direct investment shape sustainable development? Foreign direct investment is one of the main drivers for the transfer of polluting industries. With this in mind, the main objective of this research is to identify the role played by foreign direct investment (flow and stock), globalisation (*de jure* and *de facto*), corruption and regulatory quality in environmental degradation and sustainable development. To accomplish this objective, and to link the relationships under analysis to the level of development, a comparison between a group of developing countries and a group of developed ones was performed. The results confirm the suitability of the division of the countries by revealing various effects. This analysis was conducted from 1996 to 2017 and by recurring to the Autoregressive Distributed Lag model. This study proves that foreign investors play a vital role in reaching sustainable development. Measures must be implemented to eliminate the distortions that cause a company based in a country with strict environmental regulations to relocate its production to one with lax environmental regulations. However, these measures need to be combined with complementary measures that encourage developing economies to agree to a possible slowdown in their economic growth if sufficiently compensated for this reduced growth.

## 1. Introduction

Around the globe, corruption phenomena, discrepancies in environmental regulatory frameworks, and distinct economic and environmental goals among countries could undermine efforts to reduce global emissions. The Paris Agreement (2015) and The Climate and Energy Framework Agreement (2018) (https://ec.europa.eu/clima/policies/strategies/2030_en (accessed on 10 November 2020)) strengthen the necessity to combat climate change through mitigating carbon dioxide (CO_2_) emissions (used in this study as a proxy of environmental degradation, see, e.g., in [1]), with shared responsibility among countries. Environmental degradation has become one of the biggest challenges regarding economic issues, for the main reason that resources are scarce. The environment is considered within this scarcity. Although some countries are taking big steps towards sustainability, others choose to ignore the urgency of combatting climate change in favour of becoming richer.

Globalisation is considered a key feature when defining economic development and the future of the planet [2]. Globalisation is inclined to have adverse environmental effects on developing economies, although developed ones tend to experience environmental benefits from it [3]. Globalisation encompasses economic, political and social aspects [4], and Foreign Direct Investment (FDI) is a phenomenon within globalisation. The analyses of the effect of globalisation on the environment are extremely important [5], however this relationship is controversial [6] (see more in Section 2.1). On the one hand, globalisation is seen to enable countries to achieve environmental improvements [7], while on the other hand, as much of the literature recognises, globalisation is viewed as a driving force of economic growth [8] which could, consequently, be a driver of environmental degradation [7,9]. The same is noted for FDI, as it increases economic growth [10,11], but its negative effects on the environment are observed [12,13] as well. These negative effects of globalisation and FDI are external factors which have an adverse effect, negative externalities, on the environment. As stated by Rahman [14], economic growth directly affects CO_2_ emissions, as an increase in output requires more energy consumption which could lead to more environmental degradation (mainly if countries have a lower share of renewables).

Globalisation (also FDI) provides an effective framework for the transfer of polluting industries from developed to developing countries. The relocation of dirty industries phenomenon clashes with the sustainable development goals of the United Nations (such as Goal 10: Reduced Inequalities, Goal 13: Climate Action and Goal 17: Partnerships). The greatest concern is that emissions from polluting industries are transferred to the host country (commonly developing countries), maintaining, or even increasing, the global level of environmental degradation. Considering that the literature points to the relocation of pollutant industries to developing countries with permissive environmental regulation and weak exigence of governance [15], an empirical analysis of regulatory quality and corruption as governance indicators associated with this phenomenon is relevant.

Even though some researchers are investigating the role of FDI and globalisation on environmental degradation, few are using empirical evidence directed to sustainable development. Sustainable development (measured in this study by the Index of Sustainable Economic Welfare) has been a topic of discussion in diverse fields for several years. However, measuring sustainable development is quite difficult, as it must be measured by incorporating three main dimensions: social, economic and environmental (see more in Section 3.1.1). Bearing this in mind, an indicator such as the Index of Sustainable Economic Welfare (ISEW), which considers social, economic and environmental aspects, has an advantage when compared with other indicators (see more in Section 2.3). Globalisation may be considered unsustainable [16], and investigating the role of FDI on recipient countries’ sustainable development depends on the type of FDI, the kind of recipient country and their ecological footprint [17].

The analysis of FDI flow and stock in the same study is scarce in the literature [18]. To address this gap, FDI is analysed through the inward and outward FDI flow and stock to assess the effects of the relocation of pollutant industries. It is not expected that inward FDI flow and stock will have the same role as outward FDI. Therefore, it is expected that inward FDI flow and stock will have more incidence in developing countries than outward FDI. Besides being scarce in the literature, the analysis of this measurement of FDI might avoid biased results and misinterpretation. Therefore, this study innovates by analysing inward and outward FDI flow and stock to give robust empirical evidence of both the effects on environmental degradation and sustainable development due to the relocation of polluting industries.

This study aims to provide measures to combat environmental degradation without compromising sustainable development. Overall, this research innovates by providing an all-embracing analysis of the role of globalisation [19] (economic, social and political *de jure* and *de facto*) and FDI (inward and outward FDI flow and stock) on environmental degradation and sustainable development for two groups of countries: developed and developing. In light of this, the central question is “Are globalisation and FDI contributing to environmental degradation, thus compromising sustainable development?” In order to answer the central research question, this paper analyses the role of each dimension and measure of globalisation and inward and outward FDI, flow and stock, on a group of 17 developed countries and a group of 14 developing ones, by performing a comparison between them. Moreover, this paper analyses the role of corruption and regulatory quality on the relocation of polluting industries. These governance indicators were chosen as they are considered in the literature as motivation for the transfer of polluting industries. In a context of energy sources substitution and taking into account the resources widely used by FDI, the sources composition is analysed by considering both fossil fuels and renewable energy sources. This analysis is performed from the longest time period available from 1996 to 2017. Although FDI is considered a long-run phenomenon [20], the transfer of technology to developing countries could instantaneously impact the environment. With this in mind, using a methodology that permits the distinction of effects between the short- and long-run is necessary. The Autoregressive Distributed Lag (ARDL) model is most suitable in this case (see more in Section 3.2). The main findings reveal the requirement to disaggregate overall components, such as globalisation and FDI, by exposing their different effects on CO_2_ emissions and sustainable development in both groups of countries. Furthermore, governance indicators expose a relevant and distinct role in the countries under analysis, by analysing the regulatory quality and the control of corruption.

Hereinafter, this paper is structured as follows. In Section 2, a debate of the literature about the central topics of globalisation, FDI and sustainable development is given. The third section presents the data, details about ISEW calculation and the methods used in this paper. Section 4 reveals the results. Last, Section 5 and Section 6 contain a discussion and the conclusions of the main results, respectively.

## 2. Literature Background

The debate section is divided into three subsections aiming to provide an organised literature review. The first subsection contains a review of globalisation literature. The second provides a summary of the FDI–environment nexus literature. Last, the third subsection presents a literature review about sustainable development and the most usual indicators to measure it.

### 2.1. Globalisation

Globalisation is considered a useful tool helping individuals and economies trade goods and services and allowing countries to share their public policies and culture. However, it has an influential role on production, consumption, the environment and human behaviour. Consequently, it represents varied challenges in the economic, energy and environmental performance areas. The outcomes of globalisation on environmental degradation have been a focus of attention for researchers in recent times [1,4,21,22,23,24,25,26,27] and have revealed that their relationship is far from consensus [6]. On the one hand, globalisation can promote environmental degradation mitigation through the transfer of clean and green technology or by improving and sharing global ecological awareness. Beyond that, it is expected that globalisation contributes to improving countries’ welfare [7]. On the other hand, the deterioration of the environment by globalisation occurs through the stimulation of trade openness and by inducing the relocation of pollutant industries from developed to developing countries, provoking increased resources use. Through the relocation of pollutant industries, globalisation prompts a split of production from consumption, and the appropriate use of natural resources becomes a challenge with increasing globalisation [28].

Throughout the years, diverse globalisation indexes have been proposed. One that stands out is the KOF globalisation index, first presented by Dreher [29], and later improved by Dreher et al. [30]. Constituted by three dimensions, namely, economic, social and political, this index has become the most used globalisation measurement in the literature [31]. This index allows the analysis of globalisation using the KOF overall globalisation index [32] or through the economic, political and social globalisation indexes [33,34,35]. However, the use of these different measurements of globalisation could lead to different results. The studies developed by Suki et al. [36] support both the harmful and beneficial effects of globalisation on the environment. The analyses of the economic, social and political globalisation indexes reveal that economic globalisation increases environmental degradation, and political and social globalisation mitigate it. However, when analysing the overall globalisation index, a damaging effect on the environment was revealed. In light of this, a consensus about the effect of globalisation on the environment may not occur due to the measurement of globalisation used, see, e.g., in [36].

Considering that globalisation is an all-embracing and complex concept, using a proxy or an aggregate index, instead of a comprehensive analysis, could provide results that do not entirely correspond to reality. Recently, and considering the increasing complexity of reality, the KOF globalisation index was revisited by Gygli et al. [31], introducing two new measures of globalisation: *de jure* and *de facto*. Gygli et al. [31] provide a complete definition of each globalisation dimension, each measure and the respective subdimensions. This new measurement of globalisation allows a complex and extensive analysis of globalisation and avoids biased findings resulting from the use of aggregate indexes [37]. Even though the analysis of the new measures of globalisation is scarce in the literature [19,26,38], its advantages have already been revealed [37]. Briefly, distinguishing globalisation into *de jure* and *de facto* aspects is considered crucial to discern the environmental impacts of globalisation [39]. It is expected that each dimension and measure of globalisation has distinct effects on environmental degradation and sustainable development, in both nature and magnitude. Furthermore, it is also predicted that globalisation has different impacts in developed comparatively to in developing countries.

### 2.2. Foreign Direct Investment

The nexus between FDI and the environment has positive and negative externalities. Demena and Afesorgbor [18] provide a meta-analysis of the effects of FDI on the environment. There are two main hypotheses empirically tested in this subject: the Pollution Halo and the Pollution Haven Hypotheses. The Pollution Halo hypothesis posits that FDI inflows preserve the environment by reducing emissions [18,40,41,42,43]. The transference of advanced and eco-friendly technologies is the key driver for this [44]. Whereas, when FDI increases pollution, the Pollution Haven Hypothesis is supported [45,46,47,48]. Xie et al. [49] analysed the direct and indirect/spillover effects of FDI on the environment, obtaining validity for both the Pollution Haven Hypothesis, through direct effect, and the Pollution Halo Hypothesis, through spillover effect.

According to Balsalobre et al. [50], the income level of countries influences the environment mainly through three effects: scale, structural or composition, and technique. As FDI could increase income [51], these effects are also considered in the literature about the FDI–Environment nexus [52]. The scale effect is confirmed when FDI inflows increase production, thus raising energy consumption and, consequently, pollution. The structural/composition effect depends on the comparative advantages of an economy [53]. For instance, the impact of FDI differs from an economy specialised in the agriculture sector or the services sector because this transition tends to become less pollutant [50]. Last, the technique effect captures the effect of the diffusion and adoption of improved techniques. Considering all these points, it is possible to claim that the characteristics of recipient countries have great relevance to defining the impact of FDI on environmental degradation.

Nevertheless, as stated by Adeel-Farooq et al. [54], the source country of FDI also determines whether the effects are positive or negative on the recipient country’s environment. Therefore, the impact of FDI on the environment is also defined by what the source country of FDI is seeking. According to Hao et al. [55], this is disaggregated into three categories: resource-seeking, market-seeking and technology/efficiency-seeking. Resource-seeking consists of companies investing in countries with an abundance of resources (human or natural), while market-seeking denotes the search for countries with market potential. Technology-seeking comprises countries entering into high-technology foreign markets to obtain more knowledge and have easier access to technology. Among these three could emerge one more category: the search for a country with lower environmental standards to avoid environmental taxes. This category could encourage developing countries wishing to attract FDI to relax their environmental standards to gain a comparative advantage in pollution-intensive industries [11,56]. Indeed, environmental regulation plays a relevant role in defining the effect of FDI on the environment of recipient countries [41,57]. Under these circumstances, the favourable conditions for the transfer of polluting industries become united: lower environmental standards allow the relocation of polluting industries from developed to developing countries, benefiting both groups of countries, but undermining the environment worldwide.

Regarding the framework under the impact of FDI on the environment, and as both developed and developing countries are under analysis, one expects that outward FDI will impact mainly on developed countries. Moreover, inward FDI (both stock and flow) is predicted to increase emissions in developing countries while decreasing it in developed ones, thus sustaining the Pollution Haven Hypothesis.

### 2.3. Sustainable Development and Economic Growth

Increasing Gross Domestic Product (GDP) is one of the main goals of countries worldwide, but this objective may hamper sustainable development targets. Besides being powerless to measure welfare and sustainability [58,59,60,61], the goal of growing GDP can have a backlash on human wellbeing [59]. To measure sustainable development, it is crucial to incorporate the three main dimensions of sustainability: economic efficiency, social cohesion and environmental responsibility [62,63]. This is one of the main limitations found across the literature where, generally, sustainable development is measured through the use of indicators such as The Human Development Indicator [64,65] and The Ecological Footprint [17,66], that is, measured as separate indicators [58]. Frugoli et al. [67] compared 10 indices taken from the literature, and recently Kwatra et al. [68] developed a critical perspective of the evolution of sustainable development indices. The Index of Sustainable Economic Welfare (ISEW), brought into use around 1989 by Daly and Cobb [69] (lately improved in 1994 by Cobb and Cobb [70]), is a measure with a more holistic social reporting system of sustainable development than GDP.

The ISEW was introduced in the literature not as a substitute for GDP, but as an additional indicator to complement any analysis of the state of an economy [58]. Lawn [71] provides a comprehensive comparison between the ISEW and other indicators, analysing each component of them. Marques et al. [60] state that countries need to increase their productivity, but this implies future costs, the so-called defensive costs. The ISEW has the advantage of considering the three crucial defensive costs by using variables such as consumption, income inequality and natural resource depletion [60]. These are the typical defensive costs considered in the ISEW computation. They intend to reduce discrepancies in the results of the literature and make it easier to compare countries [61]. Bleys and Whitby [72] present some barriers and opportunities for ISEW calculation.

Briefly, the ISEW allows current and future wellbeing analysis, which is the keystone to sustainable development, ensuring the satisfaction of future generations’ necessities. To the best of our knowledge, the ISEW is considered a useful measure of sustainable development [73]. This indicator is currently under use in the literature, mainly in energy economics literature, such as energy–growth nexus for several areas, for instance, American countries [74], Asian countries [59], Ecuador [75], Europe [61,76], Spain [73] and Sub-Saharan countries [77]. Furthermore, it is also used in food consumption literature [78]. A study developed by Sánchez et al. [75] provides the theories of sustainable development, also explaining each component included in the ISEW calculation. As a result of the predicted impact of FDI on environmental degradation, it is expected that FDI reduces sustainable development in developing countries, and increases it in developed ones, both as inward flow and stock.

## 3. Data and Methodology

### 3.1. Data

The relocation of polluting industries is associated with contrasting features among the home and the host country. In light of this, this research aims to analyse two groups of countries with distinct features, such as level of development, income, globalisation and regulatory quality. The countries under analysis are categorised as developed and developing countries by the United Nations [79]. Categorised as developed economies, namely, Australia, Austria, Belgium, Canada, Finland, France, Germany, Ireland, Japan, Netherlands, New Zealand, Norway, Slovakia, Slovenia, Sweden, Switzerland and the United States, these countries have high levels of income, globalisation and strict environmental regulation. Categorised as developing economies, namely, Algeria, Botswana, Cameroon, Côte d’Ivoire, Egypt, Gabon, Kenya, Madagascar, Malawi, Mauritania, Morocco, Nigeria, South Africa and Tunisia, these countries have low levels of income: globalisation and lax environmental regulation. These two groups of countries were achieved through the available data of inward and outward FDI flow and stock. Regarding the time span, data availability of the governance indicators (corruption index and regulatory quality index) and ISEW computation were the main criterion for the selection. Therefore, two time periods are analysed in order to use the most available data. The models with CO_2_ emissions as the dependent variable are estimated from 1996 to 2017, while the models with ISEW as the dependent variable are estimated from 2000 to 2017. Table 1 presents the variables under analysis.

The FDI is measured as inward and outward, both stock and flows. Based on the FDI stock definition, it measures the total level of FDI at a given point in time, while FDI flows mean the value of FDI transactions during a given period. As such, it may provide an in-depth analysis of the impacts of FDI on the environment. Both the corruption index and regulatory quality index are used following the definition provided by the World Bank proposed by Kaufmann et al. [81]. The control of corruption index denotes the public perceptions of corruption, while the regulatory quality index denotes the proficiency of the government to developed and execute policies and regulations that encourage the development of the private sector. These indexes, as defined by the World Bank, are measured on a scale ranging from −2.5 to 2.5. The negative value denotes permissive governance, which means maximum corruption and low regulatory quality, while the positive value denotes rigorous governance, which means minimum corruption and high regulatory quality. The measurement of regulatory quality within developed and developing countries is often quite different due to the lower transparency of bureaucratic indicators mainly in developing countries. Thus, it is worthwhile to note that these variables are used mainly due to the data availability to allow a comparison between countries.

#### 3.1.1. Index for Sustainable Economic Welfare (ISEW)

In contrast to GDP, the ISEW is an indicator that aims to measure sustainable economic welfare. This indicator is used as a proxy of sustainable development, see, e.g., in [61,78]. Based on economic, environmental and social dimensions, the ISEW is an aggregate indicator for present and future wellbeing. As announced by Menegaki et al. [76], welfare is an uncertain and multifaceted concept; therefore, a composite indicator is crucial to measure it. An ISEW computation starts with the personal consumption expenditure weighted for income inequality, considering the advantages that rich countries benefit from economic growth contrasted to poorer countries. To this, positive welfare magnitudes, such as public health and education expenditures, are added and negative welfare magnitudes, such as environmental costs (defensive costs), are subtracted.

The ISEW calculation process employed in this research is adopted from studies such as Menegaki et al., Marques et al., and Menegaki and Tsagarakis [76,78,82]. Equation (1) describes the ISEW analysed in the study:

(1) $$ISEW=C_{w}\;+\;G_{eh}\;+\;K_{n}\;+\;S\;-\;N\;-\;C_{s},$$

where $C_{w}$ symbolises the weighted private consumption, $G_{eh}$ means the non-defensive public expenditure, $K_{n}$ denotes the net capital growth, *S* measures the unpaid work benefit, *N* consists of the depletion of the natural environment and $C_{s}$ defines the cost of social issues.

Some limitations were faced during the ISEW calculation process, such as the lack of social costs data, as handled by Menegaki et al. and Marques et al. [76,78]. Therefore, social costs were not included in the ISEW calculation. The parameters used in the calculation process of ISEW are presented in Table 2.

Owing to a lack of observations in the GINI coefficient data for a small number of countries, the missing values were calculated through the trend of the available years, see, e.g., in [80]. For example, for Botswana, Kenya, Mozambique, South Africa and Tunisia, data on the last two years were lacking and they were calculated accordingly.

#### 3.1.2. Preliminary Analysis

A preliminary analysis to evaluate the data features was conducted. Correlation is a common concern in panel data analysis. In light of this, the coefficients of the correlation matrix were assessed. Therefore, in order to avoid correlation between the variables, specific transformations were performed, namely, *GDP*, *CO_2_* and *ISEW* were transformed into per capita (pc), and *FF* and *RES* were transformed into a percentage of primary energy consumption. Descriptive statistics of the variables under analysis for both groups of countries and both time periods are displayed in Table 3 and Table 4.

Panel data specification tests were performed, such as the cross section dependence test (CD-test). Cross-sectional dependence is a common phenomenon in panel data settings, and it can lead to inconsistent outcomes once the presence of cross section dependence can bias the results of the first-generation unit root tests. Introduced by Pesaran [83], the CD-test is displayed in Table 5 and Table 6.

As displayed in Table 5 and Table 6, the null hypothesis of cross-sectional independence is rejected, confirming the presence of cross-sectional dependence for most of the variables. Therefore, the second-generation unit root test (CIPS) proposed by Pesaran [84], which is robust in the presence of cross-sectional dependence, was performed (Table 7 and Table 8).

Considering the time periods under analysis, over which diverse events could have occurred, the suspicion of the existence of structural breaks arises in the series. In light of this, the assessment of the variables unit root was taken further and as commonly used in energy economics research, see, e.g., in [19,85,86,87] the unit root test with structural breaks Zivot and Andrews [88] was conducted. Accordingly, the results of this test corroborate the results of the second-generation unit root test (the results are not presented to preserve space but available upon request). Besides that, the break points identified might be included and tested as impulse dummy variables in the estimations to control events that might have occurred in the series.

### 3.2. Methodology

Introduced in the literature by Pesaran and Smith [89] and Pesaran et al. [90], the ARDL model has diverse characteristics that can be considered as a benefit. This model is used to accomplish the main objective of this study as it allows the analysis of the dynamics of variables, disaggregating the impacts in both the short- and long-run. Furthermore, this approach is considered flexible as it permits the use of variables with distinct integration order, stationary in level or integrated of order one, I(0) and I(1), or borderline. Besides, this model allows the control of potential events through the inclusion of dummies, additionally dealing with cointegration and endogeneity issues.

The general ARDL model is provided in Equation (2):

(2) $$\Delta \varphi_{t}=\beta_{i}+\vartheta_{i1}\Delta \theta_{t}+\sum_{p=1}^{k}\beta_{1i}\varphi_{t-p}+\sum_{p=1}^{k}\beta_{2i}\theta_{t-p}+\mu_{i,t}$$

where $\varphi_{t}$ denotes the vector of dependent variables, $\beta_{i}$ represents the intercept, $\vartheta_{i1}$ denotes the semi-elasticities, $\theta_{t}$ denotes the vector of independent variables, $\beta_{1i}$ consists of the error correction mechanism (ECM), $\beta_{2i}$ denotes the elasticities and $\mu_{i,t}$ denotes the error term.

Following the general ARDL model structure, Equation (3) represents the models estimated with the ISEW:

(3) $$\begin{array}{l} DLISEW\_PC_{it}=\alpha_{0}+\sum_{j=0}^{n}\eta_{i1}DLCO2\_PC_{it}+\sum_{j=0}^{n}\eta_{i2}DLNRES\_P_{it}+\sum_{j=0}^{n}\eta_{i3}DLRES\_P_{it}+\sum_{j=0}^{n}\eta_{i4}DFDI\_F_{it}+\\ \sum_{j=0}^{n}\eta_{i5}DFDI\_S_{it}+\sum_{j=0}^{n}\eta_{i6}DLKOFECDF_{it}+\sum_{j=0}^{n}\eta_{i7}DLKOFECDJ_{it}+\sum_{j=0}^{n}\eta_{i8}DLKOFSODF_{it}+\sum_{j=0}^{n}\eta_{i9}DLKOFSODJ_{it}+\\ \sum_{j=0}^{n}\eta_{i10}DLKOFPODF_{it}+\sum_{j=0}^{n}\eta_{i11}DLKOFPODJ_{it}+\sum_{j=0}^{n}\eta_{i12}DCORR_{it}+\sum_{j=0}^{n}\eta_{i13}DRQ_{it}+\omega_{i1}LISEW\_PC_{it-1}+\\ \omega_{i2}LCO2\_PC_{it-1}+\omega_{i3}LNRES\_P_{it-1}+\omega_{i4}LRES\_P_{it-1}+\omega_{i5}FDI\_F_{it-1}+\omega_{i6}FDI\_S_{it-1}+\omega_{i7}LKOFECDF_{it-1}+\\ \omega_{i8}LKOFECDJ_{it-1}+\omega_{i9}LKOFSODF_{it-1}+\omega_{i10}LKOFSODJ_{it-1}+\omega_{i11}LKOFPODF_{it-1}+\omega_{i12}LKOFPODJ_{it-1}+\\ \omega_{i13}CORR_{it-1}+\omega_{i14}RQ_{it-1}+\varepsilon_{it} \end{array}$$

Equation (4) represents the models estimated with CO_2_ emissions as a dependent variable:

(4) $$\begin{array}{l} DLCO2\_PC_{it}=\alpha_{0}+\sum_{j=0}^{n}\eta_{i1}DLGDP\_PC_{it}+\sum_{j=0}^{n}\eta_{i2}DLNRES\_P_{it}+\sum_{j=0}^{n}\eta_{i3}DLRES\_P_{it}+\sum_{j=0}^{n}\eta_{i4}DFDI\_F_{it}+\\ \sum_{j=0}^{n}\eta_{i5}DFDI\_S_{it}+\sum_{j=0}^{n}\eta_{i6}DLKOFECDF_{it}+\sum_{j=0}^{n}\eta_{i7}DLKOFECDJ_{it}+\sum_{j=0}^{n}\eta_{i8}DLKOFSODF_{it}+\sum_{j=0}^{n}\eta_{i9}DLKOFSODJ_{it}+\\ \sum_{j=0}^{n}\eta_{i10}DLKOFPODF_{it}+\sum_{j=0}^{n}\eta_{i11}DLKOFPODJ_{it}+\sum_{j=0}^{n}\eta_{i12}DCORR_{it}+\sum_{j=0}^{n}\eta_{i13}DRQ_{it}+\omega_{i1}LCO2\_PC_{it-1}+\\ \omega_{i2}LGDP\_PC_{it-1}+\omega_{i3}LNRES\_P_{it-1}+\omega_{i4}LRES\_P_{it-1}+\omega_{i5}FDI\_F_{it-1}+\omega_{i6}FDI\_S_{it-1}+\omega_{i7}LKOFECDF_{it-1}+\\ \omega_{i8}LKOFECDJ_{it-1}+\omega_{i9}LKOFSODF_{it-1}+\omega_{i10}LKOFSODJ_{it-1}+\omega_{i11}LKOFPODF_{it-1}+\omega_{i12}LKOFPODJ_{it-1}+\\ \omega_{i13}CORR_{it-1}+\omega_{i14}RQ_{it-1}+\varepsilon_{it} \end{array}$$

In these equations, prefix D indicates first differences, $\alpha_{0}$ exemplifies the intercept, n is the lag order, $\eta_{i}$ represents the estimated parameters in the short-run, $\omega_{i}$ denotes the estimated parameters in the long-run and $\varepsilon_{it}$ symbolises the error term.

The estimation of models with diverse explanatory variables could raise the suspicion of multicollinearity among them and consequently raise concerns regarding the confidence in the results and variables’ significance. In light of this, the variance inflation factor (VIF) statistics were assessed, revealing potential multicollinearity among *FDI_si* and *FDI_so* in the models of the developed countries. Therefore, these two variables were not estimated simultaneously, and two separate models were estimated for *FDI_si* (models I and III) and for *FDI_so* (models II and IV). Furthermore, a high correlation between *KOFSODF* and *KOFSODJ* in the model of the developing countries was noticed. Consequently, two separate models were estimated for *KOFSODF* (models V and VII) and for *KOFSODJ* (models VI and VIII). Furthermore, in the developing countries’ models, a high correlation between *CO_2__pc* and *GDP_pc* was reversed through estimation the model with *CO_2_* instead of *CO_2__pc*.

The Hausman test was employed to choose the most appropriated estimator to use. The null hypothesis under this test is that the random effects are appropriate, and the results are presented in Table 9.

The rejection of the null hypothesis displayed above supports that the fixed effects estimator is the most appropriate estimator to use. Considering that, some specification tests were conducted to assess the presence of contemporaneous correlation, namely, Pesaran [83], Frees [91] and Friedman [92] tests. The presence of heteroscedasticity and autocorrelation were assessed through Modified Wald and Wooldridge tests, respectively (Table 10).

The results from the previous tests emphasise that the Driscoll–Kraay [93] estimator is the most appropriated estimator to use, as it can deal with the presence of cross section dependence, contemporaneous correlation, first-order serial correlation and heteroskedasticity. This estimator considers cross-sectional dependence and assumes that the error structure is heteroskedastic, autocorrelated and correlated among the groups in the panel.

## 4. Results

The estimations of the ARDL model and Driscoll–Kraay estimator for both the *ISEW* and *CO_2_* as dependent variables and for both groups of countries are displayed in Table 11. Bearing in mind the results of the unit roots test with structural breaks, the break points were tested in the models. Therefore, the dummy variables in 2009 and 2010 were supported by this test and demonstrated a notable adherence to an actual event, the global recession. The dummy variable included in the CO_2_ models of developed and developing countries reflects a decline in CO_2_ emissions provoked by a decrease in economic activity during the financial crisis (2008−2009) [94]. In turn, the effect on sustainable development was, on the one hand, through the reduction in capital growth (K_n_ component of the ISEW computation) and, on the other hand, through defensive costs (N component of the ISEW computation). In developed countries, this effect was immediate, and the dummy variable was included in 2009 in the ISEW model of developed countries. While in developing countries, the effect was more evident in 2010.

Besides the conducted analyses of FDI in the disaggregated form (inward and outward FDI flow and stock) provided in Table 11, FDI was also analysed as a flow and stock, by subtracting outward from inward. By comparing the outcomes of both analyses, a higher complexity in developed countries was revealed than in developing ones. On the one hand, the similarity between models for developing countries provides further support for the main findings displayed in Table 11. On the other hand, a few changes that occurred in the developed countries’ models revealed a complexity in developed countries that must be deeply analysed. This complexity could come from the diverse purposes that FDI is looking for in the developed economies, such as comparative advantages, namely, expertise and highly qualified workforce, research and development capabilities, and harnessing economies of agglomeration and clustering. In the developing economies, the aim of FDI is more predictable, in the sense that it is associated with more evident factors, such as comparative advantage on labour factor where labour-intensive industries do not necessarily require highly qualified workers. Overall, FDI in developing economies is related to more standardised and stabilised processes, while in developed ones, FDI could be a more innovative and even disruptive investment.

The analysis of FDI in the developed economies reveals that FDI stock affects the environment contrary to the effect produced on sustainable development. Both inward and outward FDI stock produced mitigation of environmental degradation and an improvement of sustainable development, suggesting that the improvement in the ISEW occurs through the reduction of the CO_2_ emissions damage costs (a component of the depletion of the natural environment (N) on the ISEW computation). In the developing economies, the stock of FDI also increases sustainable development through inward FDI; however, it increases environmental degradation as well. In these economies, inward FDI flow is the one that produced an effect on the environment contrary to the effect produced on sustainable development, reducing environmental degradation and improving sustainable development.

The analysis of globalisation through each dimension and measure proved to be useful as well. It showed an effect on the environment contrary to the effect on sustainable development in both groups of countries. Economic globalisation *de jure* raises environmental degradation and reduces sustainable development in both groups of countries. Contrasting, social globalisation improves sustainable development in both groups of countries, together with political globalisation *de jure*, while political globalisation *de facto* is harmful to it. Concerning governance, regulation quality mitigates environmental degradation, meanwhile, corruption, which can be a business enabler and consequently increases GDP, benefits sustainable development by increasing capital growth (K_n_ of ISEW computation) in both groups of countries. It is worth highlighting that the Error Correction Model (ECM) of all models is highly significant and discloses a moderate speed of adjustment to the long-run equilibrium.

The estimations shown in Table 11 resulted from using the longest time period available. In light of this, the models were estimated for different time periods. However, in order to provide results that might be more comparable, the same preliminary procedures are executed (see Table A1 and Table A2 in Appendix A), and Table A3 in Appendix A reveals the models with CO_2_ emissions as the dependent variable, estimated from 2000 to 2017. Overall, these results are similar to the results presented in Table 11, with the exceptions of loss of statistically significance of the regulatory quality in both groups of countries as well as energy consumption from renewable energy sources only in the developed countries. These two small changes could be a consequence of the exclusion of important environmental marks, such as the Kyoto protocol, which demonstrates the relevance of its inclusion and the use of the longest time period available. All the main findings are further discussed in the following section.

## 5. Discussion

The achievement of sustainable development goals is a global objective, but some countries could be facing more barriers than others to reach this. In developing countries, renewable energy consumption is not adequate enough to shrink environmental degradation as its use remains statistically insignificant, which is also found with sustainable development. Both of these outcomes are entirely predictable. As announced by Zaidi and Saidi [95], developing countries have lower available resources to implement measures to mitigate climate changes. In developed countries, renewable energy consumption has the capability to reduce environmental degradation, which reflects the high levels of investment in renewables. However, this only occurs in the short-run, suggesting that the level of investment does not meet the rising energy demand. Regarding sustainable development, renewable energy consumption seems to be decreasing it in the long-run in developed countries. As these countries have more investment in renewables, this could inhibit economic growth when the investments are supported by the government. As sustainable development is embodied by social, economic and environmental dimensions, these costs could be affecting the economic one. Fossil fuel consumption increases environmental degradation in the short- and long-run in developed and developing countries, reflecting the urgency to reduce fossil fuel dependence and increase renewable energy consumption.

The impact of corruption could depend on the objectives of the investor. Indeed, Barassi and Zhou [96] consider that corruption could attract investment when the investor chooses the host country aiming to benefit from corruption; otherwise, corruption could inhibit investment. Thus, there are two main hypotheses in the literature that analyse the impact of corruption on investments. On the one hand, the positive effect of corruption on investments is sustained in the literature as the “Helping Hand Hypothesis”, as corruption may speed the bureaucratic processes [97]. However, this only happens when investment revenues overcome the costs of corruption [98]. On the other hand, the “Grabbing Hand hypothesis” sustains the negative effect of corruption, as the indirect taxes/bribes could discourage investment [97], hampering economic growth [99]. In this study, the control of corruption (thus diminishing corruption) expands sustainable development. As such, this group of developing countries could increase their level of sustainable development by increasing their control of corruption, which could attract more investment. Considering both hypotheses, note that corruption must not be assumed as a driver for sustainable development, as it can represent long-term adverse effects that would inhibit future investments, thus undermining the growth of economies [100].

Regulatory quality, on the one hand, stimulates sustainable development in developed countries. On the other hand, regulatory quality diminishes sustainable development in developing countries. As a result, regulatory quality in developed countries incites sustainable development, perhaps due to the reduction in defensive costs (N component of ISEW computation), as the regulatory quality reduces environmental degradation. However, while regulatory quality reduces environmental degradation in developing countries, the negative impact on investment level seems larger than the relevance of reducing defensive costs. As stated by Dartey-Baah [62], the accomplishment of sustainable development goals in developing countries depends on effective leadership. Briefly, regulatory quality has a relevant role in mitigating CO_2_ emissions: it contributes to a sharp reduction in investments in developing countries, as they are frequently chosen as recipient countries for polluting industries.

Focusing on globalisation, the results obtained provide evidence that globalisation measures can have distinct effects on environmental degradation and sustainable development, in both nature and/or magnitude. Globalisation revealed some opposite effects on environmental degradation compared with the effects on sustainable development in both groups of countries. Economic globalisation *de jure* is related to trade taxes and investment restrictions, and as stated by Rudolph and Figge [101], high economically globalised countries have a high ecological footprint. Thus, economic globalisation *de jure* increases environmental degradation and simultaneously reduces sustainable development in both groups of countries. By increasing environmental degradation, the defensive costs (N component of ISEW computation) will consequently increase, suggesting that any decrease in sustainable development could be due to an increase in defensive costs. Regarding developing economies, this effect could be explained by investment restrictions which cause the continued use of older and more polluting technologies, consequently increasing environmental degradation.

Regarding social globalisation, both *de jure* and *de facto* measures contribute to increasing sustainable development and environmental degradation in the developing economies. A possible cause for that could be explained by the fact that these countries are an international tourism destination and trade in cultural goods recipients [26]. Despite increasing environmental degradation, social globalisation contributes to sustainable development, which suggests that, although social globalisation provokes environmental degradation, the increase of capital growth offsets the increase of defensive costs. In turn, in the developed economies, social globalisation *de facto* mitigates environmental degradation. This could be explained by the easier access to information components such as the use of internet and access to reliable media that could enhance environmental awareness, as stated by Ahmed et al. [102]. The improvement that social globalisation *de facto* provokes in sustainable development might come from the reduction in the defensive costs (damage from CO_2_ emissions (N)) reflected in lower emissions.

Last, while political globalisation *de facto* contributes to environmental quality improvement, political globalisation *de jure* increases environmental degradation in the developing economies under analysis. As referred by Leal et al. [26], the mitigation effect of *de facto* measures could be explained by the dissemination of environmental policies among countries, while the positive effect of *de jure* measures could provide evidence of a lack of success on global environmental management. In developed countries, political globalisation *de facto* reduces sustainable development, while political globalisation *de jure* improves it, which could be explained by the international treaties that prompt economic development. In short, sustainable development in developed countries is prompted by social globalisation *de facto*, as it shrinks CO_2_ emissions, and by political globalisation *de jure*. In developing countries, social globalisation, both *de jure* and *de facto*, is also contributing to sustainable development, but contrary to the effect on developed countries, in developing ones, social globalisation increases environmental degradation as political globalisation *de jure*. However, political globalisation *de facto* mitigates environmental degradation in developing countries.

### 5.1. The Foreign Direct Investment Impacts

Looking carefully at the FDI stock definition, it measures the total level of FDI at a given point in time, while FDI flows mean the value of FDI transactions during a given period. As expected, FDI behaves differently in the groups of countries under analysis. The stock of FDI both inward and outward revealed opposite effects on environmental degradation compared with the effects on sustainable development in the developed economies. Regarding the mitigation of environmental degradation, inward and outward FDI stock reduce emissions and increase sustainable development in developed countries. The effect of inward FDI stock reflects more environmental awareness and that these countries are receiving environmentally friendly FDI with the transfer of efficient technologies. This is also supported by the mitigating effect of inward FDI flow on environmental degradation in the developed countries. Consequently, one observes a decrease in defensive costs (N component of ISEW computation); inward FDI stock contributes to an increase in sustainable development. In turn, the outcome of outward FDI stock is anticipated and could suggest that polluting industries have been relocated from developed countries to developing countries, transferring emissions. Nonetheless, it is also important to consider that outward FDI could represent a production transfer, thus reducing energy consumption and environmental degradation.

Inward FDI stock is damaging the developing countries’ environment, which could mean that these countries are receiving polluting industries, thus supporting the Pollution Haven Hypothesis. Therefore, inward FDI stock induces environmental degradation as it considers the industries already relocated and installed. This means that the effect of inward FDI measured as stock represents the accumulation effect of FDI on the environment [103]. Thus, this result is not unexpected in developing countries. Inward FDI flow appears to reduce CO_2_ emissions and be environmentally conscious in the long-run in developing countries, thus contributing to environmental preservation. The greater the gap between the technological level in the source country of FDI and the recipient ones, the greater the effect of the introduction of technological processes [104]. This outcome may not be a consequence of an increase in environmental stringency in developing countries, but it might suggest that source countries are no longer transferring obsolete technologies.

Regarding sustainable development in developing countries, it has been positively affected by inward FDI both as a flow and stock. Following the assumption that FDI induces economic growth, this outcome is expected. As FDI increases income and the rate of employment, then the social consequences are reduced while FDI is increasing [105]. This means that, besides contributing to economic development, FDI could improve social welfare [104]. Briefly, the attraction of foreign investors could support developing countries to reach the sustainable development goals proposed by the United Nations [63]. However, how could it be possible to suggest attracting foreign investors while inward FDI stock is contributing to environmental degradation? The answer is that the harmful effect that inward FDI stock provokes on the environment is offset by the improvement in sustainable development through the increase in economic capital. In short, the main findings provide evidence for the transfer of polluting industries from developed to developing countries, supporting the Pollution Haven Hypothesis. However, inward FDI flows bring developing countries environmentally friendly technology, revealing that they are no longer receiving obsolete and pollutant technology. It announces that FDI could be mandatory to reach sustainable development goals, mainly in developing countries, but they must receive these technologies and knowledge, and adopt them in their own production process.

The main findings expose a potential new paradigm under the analysis of the relocation of polluting industries. While inward FDI stock supports the transfer of polluting industries, inward FDI flow uncovers the indirect role of the investment in innovation in developed countries. Besides, this result also reveals the crucial role of energy consumption. Although the adoption of energy-efficient techniques through FDI could be considered as crucial to reduce emissions [106], the energy source might also be taken into account. Most of the energy consumed in developing countries comes from fossil fuels, as these countries also lower the availability of resources to invest in renewable energy projects. This means that an increase in energy consumption in developing countries will rise pollution (which is supported by the results). Thus, besides diminishing environmental regulation discrepancies among developed and developing countries, the governments of developing countries must attract investment towards renewable energy generation projects. Moreover, these countries ought to improve their governance standards by increasing the control of corruption, as they could attract more investment, also contributing to sustainable development. The investment in innovation towards Research and Development grants, for example, must be steadily encouraged in developed countries.

### 5.2. Research Gaps and Future Lines of Research

This investigation is developed under the objective of filling some gaps found in the literature. Indeed, each dimension and measure of globalisation is considered. The results reveal the suitability of conducting this disaggregation as it could have different impacts in nature and/or magnitude. Furthermore, to better discern the transfer of polluting industries from developed to developing countries, inward and outward FDI are analysed, both as flow and stock. Besides, the measurement of sustainable development by the ISEW is innovative in this subject. By analysing and comparing developed and developing countries, it was fulfilled a gap in the literature by analysing whether globalisation and FDI are harming environmental degradation and compromising sustainable development.

Environmental degradation has become one of the biggest challenges facing the planet. Resources are scarce, and the environment is considered within this scarcity. The fight against climate change is urgent, and although some countries are taking significant steps towards environmental degradation mitigation, others are ignoring this urgency. The Glasgow summit (26th United Nations Climate Change Conference of the Parties (COP26) in Glasgow), originally scheduled to take place in November 2020, has been rescheduled to November 2021 as a consequence of the current COVID-19 pandemic situation. The Paris Agreement (COP21) outlined the necessity to meet every five years to re-evaluate the current state of climate change. COP26 is the first time that parties are expected to commit to enhancing the ambition to tackle climate change. A key topic of discussion at the Glasgow summit should be the transfer of pollutant industries to developing countries. It is in these countries that the transfer has the greatest repercussions, and as a driver of environmental degradation, mechanisms to discourage it and/or compensate those host countries receiving it should be a focus.

This study suggests that future lines of research could involve conducting a similar analysis to the present one by considering other indicators of sustainable development, including social, economic and environmental aspects of sustainability. Furthermore, the use of ecological footprint as a proxy for environmental degradation could bring an interesting discussion to compare results with a sustainable development indicator, as it is used in the literature as a proxy for both environmental degradation, see, e.g., in [102], and sustainable development, see, e.g., in [17,66]. Further research could also include more governance indicators such as the rule of law and political stability, as they could influence the investment decisions of foreign investors.

## 6. Conclusions

This research was undertaken with the objective of evaluating the nature of polluting industries transfer from developed to developing countries. To do that, this study analyses the influence of FDI (measured as inward and outward FDI flow and stock) on environmental degradation. This study also aims to analyse the consequences of the phenomenon of industry relocation on sustainable development (measured through the ISEW) for both home and host countries. This research goes beyond innovating through analysing the specific effect of inward and outward FDI flow and stock on developed and developing economies; the influence of each dimension and measure of globalisation is considered as well. Additionally, governance indicators were included in the analysis. The analysis of both inward and outward FDI flow and stock, and the analysis of globalisation through the three dimensions and two measures, make it possible to go deeper into the study of the complexity of globalisation and FDI on the environment, and to the best of our knowledge, this approach is innovative.

When looking for governance indicators, the effect of corruption seems to be helpful to reach sustainability. Corruption may speed bureaucratic processes and assumes the role of business facilitator. However, corruption can represent long-term adverse effects that could be undermining the growth of economies. Regarding regulatory quality, it reduces environmental degradation in both groups of countries, which helps countries to reach sustainable development goals. In developed countries, this beneficial environmental effect is reflected in an increase in sustainable growth through the reduction of environmental costs. In the developing ones, the loss of capital growth is not offset by the reduction in environmental costs, thus reducing sustainable development. These effects evidenced the lax and undemanding environmental regulation in the developing countries analysed in the study.

The analysis of inward and outward FDI flow and stock in developed countries reveals that both improve environmental quality and sustainable development. In these countries, the reduction in emissions that consequently leads to a reduction in defensive costs (damage from CO_2_ emissions (N)) results in improved sustainable development. On the one hand, inward FDI flow and stock suggest that the investments received by developed economies have an environmental awareness background. On the other hand, the environmental mitigation effect of outward FDI stock in developed countries might suggest that emissions have been transferred through the relocation of pollutant industries to developing countries. As a result, inward FDI stock in both the short- and long-run increases environmental degradation in developing countries.

The undemanding environmental regulation in developing countries adds to the defensive costs that are subsequently offset by capital growth. Inward FDI stock provokes an improvement in sustainable development. As is evidenced by the effects of regulatory quality in developing countries, this research maintains that it is necessary to implement measures that encourage these countries to improve their environmental regulation. Notwithstanding, it is essential to compensate recipient countries for the loss of investment that may result from tightening environmental legislation.

## Figures and Tables

**Table 1:** Variables.

Variable	Description	Source
*ISEW*	Index for Sustainable Economic Welfare	Own calculation
*CO_2_*	Carbon Dioxide Emissions (MMtons)	U.S. Energy Information Administration
*FDI_fi*	Inward Foreign Direct Investment Flow (% of GDP)	UNCTADStat
*FDI_fo*	Outward Foreign Direct Investment Flow (% of GDP)	
*FDI_si*	Inward Foreign Direct Investment Stock (% of GDP)	
*FDI_so*	Outward Foreign Direct Investment Stock (% of GDP)	
*GDP*	Gross Domestic Product (constant 2010 US$)	World Bank Development Indicators
*CORR*	Control of Corruption Index	World Bank, Worldwide Governance Indicators
*RQ*	Regulatory Quality Index	
*FF*	Fossil fuels consumption (quad Btu)	U.S. Energy Information Administration
*RES*	Renewable energy consumption (quad Btu)	
*KOFECDF*	KOF Globalisation Index Economic Dimension *de Facto*	KOF Swiss Economic Institute
*KOFECDJ*	KOF Globalisation Index Economic Dimension *de Jure*	
*KOFSODF*	KOF Globalisation Index Social Dimension *de Facto*	
*KOFSODJ*	KOF Globalisation Index Social Dimension *de Jure*	
*KOFPODF*	KOF Globalisation Index Political Dimension *de Facto*	
*KOFPODJ*	KOF Globalisation Index Political Dimension *de Jure*	

**Notes:** MMtons denotes Million Metric tonnes; quad Btu denotes quadrillion British thermal unit. Interpolation technique with the average of the values from the adjacent years was employed to calculate the years of 1997, 1999 and 2001 of the governance indicators (corruption index and regulatory quality index), see e.g., in [80].

**Table 2:** Index of Sustainable Economic Welfare (ISEW) components.

Variables	Sign	Calculation Method	Source
Adjusted personal consumption with durables (C_w_)	+	PC * (1-GINI)	PC: https://data.worldbank.org/indicator/NE.CON.PRVT.CD GINI: https://dataverse.harvard.edu/dataset.xhtml?persistentId=doi:10.7910/DVN/LM4OWF
Education expenditure (G_eh_)	+	Education expenditure * 0,5	http://data.worldbank.org/indicator/NY.ADJ.AEDU.CD
Health expenditure (G_eh_)	+	Health expenditure * 0,5	https://data.worldbank.org/indicator/SH.XPD.CHEX.PC.CD
Net capital growth (K_n_)	±	GCF-CFC	FCA: http://data.worldbank.org/indicator/NE.GDI.TOTL.CD CFC: http://data.worldbank.org/indicator/NY.ADJ.DKAP.CD
Mineral depletion (N)	-		http://data.worldbank.org/indicator/NY.ADJ.DMIN.CD
Energy depletion (N)	-		http://data.worldbank.org/indicator/NY.ADJ.DNGY.CD
Forest depletion (N)	-		http://data.worldbank.org/indicator/NY.ADJ.DFOR.CD
Damage from CO_2_ emissions (N)	-		http://data.worldbank.org/indicator/NY.ADJ.DCO2.CD

**Notes:** * denotes multiplication; PC denotes Final household consumption expenditure; FCA denotes Gross capital formation; CFC denotes consumption of fixed capital. The sources were last accessed on 12 October 2020.

**Table 3:** Descriptive statistics from 1996 to 2017 (CO_2_ model).

Developed Countries					
	Mean	Std. Dev.	Min.	Max.	Obs.
*CO_2__pc*	1.08 × 10^−5^	4.23 × 10^−6^	4.75 × 10^−6^	2.08 × 10^−5^	374
*GDP_pc*	45,323.42	16,260.43	9290.189	91565.73	374
*FDI_fi*	3.9302	6.8449	−14.9729	74.7406	374
*FDI_fo*	4.0949	6.1196	−6.8562	57.7976	374
*FDI_si*	47.1199	42.3908	0.6134	315.1932	374
*FDI_so*	49.5991	51.6383	0.8444	312.0545	374
*FF_p*	70.3175	18.5059	30.1784	98.3638	374
*RES_p*	18.1771	16.8212	1.0816	69.8216	374
*CORR*	1.7084	0.5393	−0.0066	2.4650	374
*RQ*	1.4668	0.3507	0.5133	2.0980	374
*KOFECDF*	68.2951	14.7306	29.2683	92.0529	374
*KOFECDJ*	83.0661	7.3250	50.1067	94.7231	374
*KOFSODF*	81.5734	7.3341	55.3301	95.1544	374
*KOFSODJ*	84.4961	4.6587	64.9952	92.6337	374
*KOFPODF*	88.7225	7.0416	44.8963	98.0264	374
*KOFPODJ*	90.0320	9.9720	57.0964	100	374
**Developing Countries**					
	**Mean**	**Std. Dev.**	**Min.**	**Max.**	**Obs.**
*CO_2__pc*	61.737	116.7421	0.8331	481.3982	308
*GDP_pc*	3101.381	2714.604	361.0435	11,949.28	308
*FDI_fi*	2.5574	3.1981	−7.8678	28.909	308
*FDI_fo*	0.1727	0.6498	−2.6152	6.6982	308
*FDI_si*	26.6481	19.4782	0.6565	103.8824	308
*FDI_so*	4.1227	8.1729	0.0065	79.3375	308
*FF_p*	86.1203	14.8569	41.7286	99.8846	308
*RES_p*	13.7023	15	0.0533	58.2714	308
*CORR*	−0.4958	0.583	−1.4312	1.2167	308
*RQ*	−0.3579	0.4799	−1.352	0.8042	308
*KOFECDF*	49.3981	9.8315	29.858	68.8775	308
*KOFECDJ*	44.8139	10.6806	23.2961	69.5586	308
*KOFSODF*	38.3162	14.7217	7.9692	66.9936	308
*KOFSODJ*	48.1803	12.8073	17.4248	74.0204	308
*KOFPODF*	65.3888	19.6631	24.137	93.309	308
*KOFPODJ*	71.5454	14.0672	27.1605	94.1716	308

**Notes:** Max. denotes Maximum; Min. denotes Minimum; Std. Dev. denotes Standard deviation; Obs denotes Observations; pc denotes per capita.

**Table 4:** Descriptive statistics from 2000 to 2017 (ISEW model).

Developed Countries					
	Mean	Std. Dev.	Min.	Max.	Obs.
*ISEW_pc*	20,787.8	8107.95	2571.254	45,772.46	306
*CO_2__pc*	1.07 × 10^−5^	4.25 × 10^−6^	4.75 × 10^−6^	4.75 × 10^−5^	306
*FDI_fi*	3.9396	6.9368	−14.9729	74.7406	306
*FDI_fo*	4.1529	6.0808	−6.8562	57.7976	306
*FDI_si*	52.0087	44.6233	1.0296	315.1932	306
*FDI_so*	55.3134	54.9308	1.5233	312.0545	306
*FF_p*	69.9704	18.5276	30.1784	98.3638	306
*RES_p*	18.4964	16.7479	1.6199	69.8216	306
*CORR*	1.7074	0.5397	−0.0066	2.465	306
*RQ*	1.4845	0.3385	0.5305	2.0980	306
*KOFECDF*	70.2098	13.9295	29.8409	92.0529	306
*KOFECDJ*	83.2217	6.0357	62.5938	94.7231	306
*KOFSODF*	83.0283	6.6071	59.5395	95.1544	306
*KOFSODJ*	85.7722	3.3440	73.927	92.6337	306
*KOFPODF*	89.1547	6.3996	70.3025	98.0264	306
*KOFPODJ*	90.8493	9.3272	64.4206	100	306
**Developing Countries**					
	**Mean**	**Std. Dev.**	**Min.**	**Max.**	**Obs.**
*ISEW_pc*	1016.448	740.7478	7.1543	3345.461	252
*CO_2__pc*	1.69 × 10^−6^	2.23 × 10^−6^	5.81 × 10^−8^	9.45 × 10^−6^	252
*FDI_fi*	2.9123	3.3016	−1.7194	28.909	252
*FDI_fo*	0.1791	0.6995	−2.6152	6.6982	252
*FDI_si*	28.9467	19.7936	1.5335	103.8824	252
*FDI_so*	4.2206	8.703	0.0314	79.3375	252
*FF_p*	86.1318	15.0328	41.7286	99.8846	252
*RES_p*	13.6960	15.1698	0.0935	58.2714	252
*CORR*	−0.5044	0.5736	−1.4312	1.2167	252
*RQ*	−0.379	0.4804	−1.352	0.8042	252
*KOFECDF*	49.8868	9.9283	29.858	68.8775	252
*KOFECDJ*	45.6768	10.6771	23.2961	69.5586	252
*KOFSODF*	40.6245	14.088	9.9558	66.9936	252
*KOFSODJ*	50.8484	11.5413	25.8745	74.0204	252
*KOFPODF*	67.4178	18.8854	25.4038	93.309	252
*KOFPODJ*	73.5866	12.5386	43.4034	94.1716	252

**Notes:** Max. denotes Maximum; Min. denotes Minimum; Std. Dev. denotes Standard deviation; Obs denotes Observations; pc denotes per capita.

**Table 5:** Cross section dependence test from 1996 to 2017 (CO_2_ model).

Developed Countries				Developing Countries			
	CD-Test	Corr	Abs (corr)		CD-Test	Corr	Abs (corr)
*LCO_2__pc*	35.87 ***	0.656	0.663	*LCO_2__pc*	25.96 ***	0.58	0.721
*LGDP_pc*	51.56 ***	0.943	0.943	*LGDP_pc*	20.26 ***	0.453	0.644
*FDI_fi*	9.34 ***	0.171	0.242	*FDI_fi*	2.73 ***	0.061	0.199
*FDI_fo*	7.06 ***	0.129	0.235	*FDI_fo*	1.81 *	0.04	0.227
*FDI_si*	28.13 ***	0.514	0.643	*FDI_si*	18.52 ***	0.414	0.584
*FDI_so*	31.97 ***	0.585	0.674	*FDI_so*	9.08 ***	0.203	0.661
*LNRES_p*	19.22 ***	0.351	0.599	*LNRES_p*	−2.07 **	−0.046	0.399
*LRES_p*	24.59 ***	0.449	0.563	*LRES_p*	−1.47	−0.033	0.404
*CORR*	0.89	0.016	0.319	*CORR*	−1.67 *	−0.037	0.35
*RQ*	3 ***	0.055	0.394	*RQ*	8.76 ***	0.196	0.362
*LKOFECDF*	30.11 ***	0.551	0.706	*LKOFECDF*	0.39	0.009	0.399
*LKOFECDJ*	17.42 ***	0.319	0.534	*LKOFECDJ*	8.19 ***	0.183	0.363
*LKOFSODF*	52.57 ***	0.961	0.961	*LKOFSODF*	42 ***	0.939	0.939
*LKOFSODJ*	46.29 ***	0.846	0.846	*LKOFSODJ*	41.89 ***	0.936	0.936
*LKOFPODF*	10.88 ***	0.199	0.381	*LKOFPODF*	23.54 ***	0.526	0.534
*LKOFPODJ*	38.81 ***	0.71	0.813	*LKOFPODJ*	39.79 ***	0.889	0.889

**Notes:** *, **, *** denotes statistical significance level at 10%, 5% and 1%, respectively; prefix L denotes Logarithm.

**Table 6:** Cross section dependence test from 2000 to 2017 (ISEW model).

Developed Countries				Developing Countries			
	CD-Test	Corr	Abs (corr)		CD-Test	Corr	Abs (corr)
*ISEW_pc*	41.9 ***	0.847	0.847	*ISEW_pc*	30.47 ***	0.753	0.753
*CO_2__pc*	35.88 ***	0.725	0.727	*CO_2__pc*	5.06 ***	0.125	0.486
*FDI_fi*	9.63 ***	0.195	0.264	*FDI_fi*	1.38	0.034	0.207
*FDI_fo*	5.52 ***	0.112	0.243	*FDI_fo*	1.75 *	0.043	0.239
*FDI_si*	23.17 ***	0.468	0.536	*FDI_si*	13.96 ***	0.345	0.517
*FDI_so*	23.26 ***	0.470	0.622	*FDI_so*	8.95 ***	0.221	0.642
*FF_p*	19.53 ***	0.395	0.598	*FF_p*	−1.93 *	−0.048	0.436
*RES_p*	25.32 ***	0.512	0.6	*RES_p*	−1.35	−0.033	0.448
*CORR*	1.07	0.022	0.345	*CORR*	−0.85	−0.021	0.353
*RQ*	1.2	0.024	0.412	*RQ*	9.58 ***	0.237	0.430
*KOFECDF*	20.16 ***	0.407	0.705	*KOFECDF*	0.46	0.011	0.392
*KOFECDJ*	16.21 ***	0.328	0.5	*KOFECDJ*	0.79	0.02	0.308
*KOFSODF*	46.75 ***	0.945	0.945	*KOFSODF*	36.91 ***	0.912	0.912
*KOFSODJ*	31.73 ***	0.641	0.641	*KOFSODJ*	34.73 ***	0.858	0.858
*KOFPODF*	5.37 ***	0.109	0.401	*KOFPODF*	15.72 ***	0.388	0.418
*KOFPODJ*	34.22 ***	0.692	0.74	*KOFPODJ*	36.33 ***	0.898	0.898

**Notes:** *, *** denotes statistical significance level at 10% and 1%, respectively; prefix L denotes Logarithm.

**Table 7:** Second-generation unit root test (CIPS) from 1996 to 2017 (CO_2_ model).

	Developed Countries		Developing Countries	
	Without Trend	With Trend	Without Trend	With Trend
*LCO2_pc*	−2.944 ***	−2.14 **	0.613	−0.841
*DLCO2_pc*	−11.039 ***	−9.149 ***	−11.984 ***	−10.258 ***
*LGDP_pc*	0.329	1.957	2.685	3.988
*DLGDP_pc*	−6.549 ***	−4.751 ***	−6.26 ***	−6.195 ***
*FDI_fi*	−8.15 ***	−7.735 ***	−5.857 ***	−5.783 ***
*DFDI_fi*	−17.031 ***	−15.634 ***	−13.276 ***	−11.878 ***
*FDI_fo*	−7.120 ***	−7.154 ***	−3.837 ***	−4.761 ***
*DFDI_fo*	−15.448 ***	−13.731 ***	−14.191 ***	−12.454 ***
*FDI_si*	−0.081	3.848	0.835	2.581
*DFDI_si*	−9.893 ***	−9.111 ***	−7.556 ***	−6.287 ***
*FDI_so*	0.304	3.185	6.532	3.713
*DFDI_so*	−8.160 ***	−7.123 ***	−5.196 ***	−3.958 ***
*LFF_p*	−3.677 ***	−2.48 ***	0.785	−1.257
*DLFF_p*	−13.481 ***	−12.046 ***	−11.733 ***	−10.755 ***
*LRES_p*	−3.862 ***	−3.214 ***	1.1	0.906
*DLRES_p*	−14.63 ***	−13.908 ***	−11.728 ***	−10.515 ***
*CORR*	0.525	0.636	0.234	2.23
*DCORR*	−9.524 ***	−6.854 ***	−6.340 ***	−4.785 ***
*RQ*	−0.645	1.475	−1.239	0.051
*DRQ*	−8.281 ***	−6.315 ***	−8.073 ***	−5.958 ***
*LKOFECDF*	−2.438 ***	−1.671 **	−0.293	−1.792 **
*DLKOFECDF*	−8.107 ***	−6.122 ***	−10.32 ***	−8.413 ***
*LKOFECDJ*	−0.831	0.435	−1.049	−1.07
*DLKOFECDJ*	−12.93 ***	−11.854 ***	−10.933 ***	−9.074 ***
*LKOFSODF*	−2.941 ***	−1.137	−1.805 **	−2.16 **
*DLKOFSODF*	−12.925 ***	−11.6 ***	−12.089 ***	−10.317 ***
*LKOFSODJ*	−3.582 ***	−2.429 ***	−1.945 **	−0.553
*DLKOFSODJ*	−11.196 ***	−10.076 ***	−9.548 ***	−7.961 ***
*LKOFPODF*	−3.942 ***	−1.55 *	−4.018 ***	−1.741 **
*DLKOFPODF*	−10.943 ***	−9.326 ***	−10.313 ***	−9.237 ***
*LKOFPODJ*	−3.052 ***	−2.888 ***	−5.477 ***	−5.869 ***
*DLKOFPODJ*	−11.055 ***	−10.553 ***	−13.519 ***	−11.983 ***

**Notes:** *, **, *** denotes statistical significance level at 10%, 5% and 1%, respectively; prefix D denotes Differences; prefix L denotes Logarithm.

**Table 8:** Second-generation unit root test (CIPS) from 2000 to 2017 (ISEW model).

	Developed Countries		Developing Countries	
	Without Trend	With Trend	Without Trend	With Trend
*LISEW_pc*	−0.417	0.385	−1.174	−0.436
*DLISEW_pc*	−7.361 ***	−4.160 ***	−6.369 ***	−5.002 ***
*LCO2_pc*	−3.065 ***	−0.995	0.838	−2.682 ***
*DLCO2_pc*	−8.052 ***	−5.553 ***	−9.340 ***	−8.045 ***
*FDI_fi*	−7.593 ***	−6.8 ***	−4.105 ***	−3.799 ***
*DFDI_fi*	−15.068 ***	−12.919 ***	−11.165 ***	−9.489 ***
*FDI_fo*	−7.482 ***	−6.073 ***	−2.136 **	−3.305 ***
*DFDI_fo*	−13.476 ***	−10.870 ***	−10.858 ***	−9.056 ***
*FDI_si*	−1.515 *	1.702	0.656	2.539
*DFDI_si*	−7.736 ***	−7.394 ***	−4 ***	−3.301 ***
*FDI_so*	0.274	2.531	4.661	1.908
*DFDI_so*	−5.599 ***	−3.818 ***	−4.755 ***	−2.968 ***
*LFF_p*	−2.627 ***	−2.219 **	−0.805	−0.358
*DLFF_p*	−11.802 ***	−9.625 ***	−9.081 ***	−8.636 ***
*LRES_p*	−3.806 ***	−3.954 ***	1.047	−0.612
*DLRES_p*	−13.078 ***	−11.109 ***	−10.663 ***	−10.055 ***
*CORR*	0.123	0.562	−0.318	1.441
*DCORR*	−8.061 ***	−5.491 ***	−5.126 ***	−3.399 ***
*RQ*	−1.338 *	−0.067	−1.678 **	−0.152
*DRQ*	−7.611 ***	−6.928 ***	−6.751 ***	−4.862 ***
*LKOFECDF*	−1.140	−0.912	0.186	−0.205
*DLKOFECDF*	−8.653 ***	−7.041 ***	−8.094 ***	−6.586 ***
*LKOFECDJ*	−1.962 **	−0.212	−0.463	0.339
*DLKOFECDJ*	−9.598 ***	−8.882 ***	−7.761 ***	−5.984 ***
*LKOFSODF*	−3.234 ***	−2.397 ***	−2.477 ***	−3.652 ***
*DLKOFSODF*	−10.151 ***	−7.615 ***	−10.81 ***	−10.244 ***
*LKOFSODJ*	−4.456 ***	−2.180 **	−0.69	−0.096
*DLKOFSODJ*	−9.994 ***	−9.122 ***	−6.566 ***	−5.79 ***
*LKOFPODF*	−0.681	1.597	−1.262	−1.363 *
*DLKOFPODF*	−6.973 ***	−6.662 ***	−7.802 ***	−6.171 ***
*LKOFPODJ*	−2.469 ***	−1.685 **	−2.941 ***	−4.743 ***
*DLKOFPODJ*	−8.551 ***	−7.307 ***	−10.451 ***	−8.923 ***

**Notes:** *, **, *** denotes significance level at 10%, 5% and 1%, respectively; prefix D denotes Differences; prefix L denotes Logarithm.

**Table 9:** Hausman test and F-test.

	Developed Countries			
	**I-CO_2_ Model with FDI_si**	**II-CO_2_ Model with FDI_so**	**III-ISEW Model with FDI_si**	**IV-ISEW Model with FDI_so**
F-test	3.59 ***	3.56 ***	3 ***	2.38 ***
Hausman test	50.93 ***	50.62 ***	42.75 ***	35.04 ***
	**Developing Countries**			
	**V-CO_2_ Model with KOFSODF**	**VI-CO_2_ Model with KOFSODJ**	**VII-ISEW Model with KOFSODF**	**VIII-ISEW Model with KOFSODJ**
F-test	2.9 ***	2.89 ***	7.05 ***	9.36 ***
Hausman test	34.45 ***	34.35 ***	66.49 ***	79.91 ***

**Notes:** *** denotes significance level at 1%.

**Table 10:** Specification tests.

	Developed Countries			
	**I-CO_2_ Model with FDI_si**	**II-CO_2_ Model with FDI_so**	**III-ISEW Model with FDI_si**	**IV-ISEW Model with FDI_so**
Pesaran test	8.636 ***	8.775 ***	16.806 ***	15.198 ***
Frees test	0.549 ***	0.579 ***	2.217 ***	1.997 ***
Fiedman test	66.108 ***	66.411 ***	105.232 ***	100.286 ***
Modified Wald test	320.32 ***	361.58 ***	44.40 ***	66.98 ***
Wooldrige test	100.426 ***	87.545 ***	118.034 ***	95.314 ***
	**Developing Countries**			
	**V-CO_2_ Model with KOFSODF**	**VI-CO_2_ Model with KOFSODJ**	**VII-ISEW Model with KOFSODF**	**VIII-ISEW Model with KOFSODJ**
Pesaran test	1.999 **	1.899 *	4.513 ***	4.792 ***
Frees test	0.041	−0.005	1.027 ***	0.837 ***
Fiedman test	34.03 ***	34.141 ***	39.653 ***	43.227 ***
Modified Wald test	176.37 ***	187.71 ***	706.11 ***	650.51 ***
Wooldrige test	55.846 ***	52.965 ***	16.456 ***	14.367 ***

**Notes:** ***, **, * denotes significance level at 1%, 5% and 10%, respectively.

**Table 11:** Estimations of Autoregressive Distributed Lag (ARDL) model.

	Developed Countries				Developing Countries				
	CO_2_ Model		ISEW Model		ISEW Model			CO_2_ Model	
	I-FDI_si	II-FDI_so	III-FDI_si	IV-FDI_so	VII-KOFSODF	VIII-KOFSODJ		V-KOFSODF	VI-KOFSODJ
*DLGDP_pc*	0.4421 ***	0.4515 ***					*DLGDP_pc*		
*DLCO2_pc*							*DLCO2*		
*DFDI_fi*	−0.0006 **	−0.0006 **			0.015 ***	0.0128 ***	*DFDI_fi*		
*DFDI_fo*							*DFDI_fo*		
*DFDI_si*							*DFDI_si*	0.0019 **	0.0013 *
*DFDI_so*							*DFDI_so*	−0.0051 ***	−0.0048 ***
*DLFF_p*	0.8718 ***	0.8698 ***					*DLFF_p*	1.5867 ***	1.6863 ***
*DLRES_p*	−0.0267 **	−0.0282 **					*DLRES_p*		
*DCORR*			0.0976 **	0.0892 **	0.2731 **		*DCORR*		
*DRQ*			0.0693 **		−0.2738 *		*DRQ*	−0.0750 **	−0.0677 *
*DLKOFECDF*			−0.8967 ***	−0.9049 ***			*DLKOFECDF*		
*DLKOFECDJ*						−0.5456 ***	*DLKOFECDJ*	0.2732 ***	0.1932 ***
*DLKOFSODF*			0.9839 ***	0.837 **	0.4057 **		*DLKOFSODF*		
*DLKOFSODJ*						0.9309 ***	*DLKOFSODJ*		
*DLKOFPODF*							*DLKOFPODF*		
*DLKOFPODJ*							*DLKOFPODJ*		
*LISEW_pc (−1)*			−0.1407 ***	−0.1401 ***	−0.7447 ***	−0.7586 ***	*LISEW_pc (−1)*		
*LCO2_pc (−1)*	−0.1927 ***	−0.1986 ***	0.2863 ***	0.2597 ***	0.3267 **	0.2485 **	*LCO2 (−1)*	−0.2275 ***	−0.2214 ***
*LGDP_pc (−1)*							*LGDP_pc (−1)*		
*FDI_fi (−1)*							*FDI_fi (−1)*	−0.0036 **	−0.0032 **
*FDI_fo (−1)*							*FDI_fo (−1)*		
*FDI_si (−1)*	−0.00011 **		0.0012 ***		0.008 ***	0.0051 ***	*FDI_si (−1)*	0.00092 *	
*FDI_so (−1)*		−0.00013 **		0.00068 ***			*FDI_so (−1)*		
*LFF_p (−1)*	0.2861 ***	0.2908 ***					*LFF_p (−1)*	0.4846 ***	0.6031 ***
*LRES_p (−1)*			−0.0496 ***	−0.0658 ***			*LRES_p (−1)*		
*CORR (−1)*			0.1028 ***	0.0895 ***	0.3039 **	0.2419 **	*CORR (−1)*		
*RQ (−1)*	−0.0156 **	−0.0177 **					*RQ (−1)*	−0.0411 *	
*LKOFECDF (−1)*							*LKOFECDF (−1)*		
*LKOFECDJ (−1)*	0.0971 ***	0.0893 ***	−0.5117 ***	−0.5396 ***	−0.4261 *	−0.5397 **	*LKOFECDJ (−1)*	0.12 *	
*LKOFSODF (−1)*	−0.1115 ***	−0.1014 **			0.7076 ***		*LKOFSODF (-1)*	0.1063 ***	
*LKOFSODJ (−1)*						1.5626 ***	*LKOFSODJ (−1)*		0.1636 ***
*LKOFPODF (−1)*			−0.8895 ***	−0.8307 ***			*LKOFPODF (−1)*	−0.1081 **	−0.0871 **
*LKOFPODJ (−1)*			1.1089 ***	1.1739 ***			*LKOFPODJ (−1)*	0.2089 ***	0.284 ***
*C*	−3.3438 ***	−3.438 ***	5.8755 ***	5.2215 ***	8.5173 ***	4.4613 *	*C*	−2.8327 ***	−3.5374 ***
*Dum_2009*	−0.0281 ***	−0.0279 ***	−0.1923 ***	−0.2006 ***			*Dum_2009*	−0.0948 ***	−0.0962 ***
*Dum_2010*					0.0806 **	0.14405 ***	*Dum_2010*		
**Elasticities**									
*LCO2_pc (−1)*			2.0346 *	1.8545 *	0.4387 ***	0.3276 **	*LCO2 (−1)*		
*LGDP_pc (−1)*							*LGDP_pc (−1)*		
*FDI_fi (−1)*							*FDI_fi (−1)*	−0.0159 ***	−0.0144 ***
*FDI_fo (−1)*							*FDI_fo (−1)*		
*FDI_si (−1)*	−0.00058 **		0.0088 **		0.0107 ***	0.0067 ***	*FDI_si (−1)*	0.004 **	
*FDI_so (−1)*		−0.00064 ***		0.0049 **			*FDI_so (−1)*		
*LFF_p (−1)*	1.4851 ***	1.4641 ***					*LFF_p (−1)*	2.1307 ***	2.7238 ***
*LRES_p (−1)*			−0.3522 ***	−0.4698 ***			*LRES_p (−1)*		
*CORR (−1)*			0.7308 ***	0.6391 **	0.4081 **	0.3189 **	*CORR (−1)*		
*RQ (−1)*	−0.0811 **	−0.08887**					*RQ (−1)*	−0.1806 **	
*LKOFECDF (−1)*							*LKOFECDF (−1)*		
*LKOFECDJ (−1)*	0.5039 ***	0.4494 ***	−3.6371 *	−3.8532 *	−0.5721 **	−0.7115 ***	*LKOFECDJ (−1)*	0.5275 **	
*LKOFSODF (−1)*	−0.5787 ***	−0.5106 ***			0.9501 ***		*LKOFSODF (−1)*	0.4671 ***	
*LKOFSODJ (−1)*						2.06 ***	*LKOFSODJ (−1)*		0.7387 ***
*LKOFPODF (−1)*			−6.3223 **	−5.9316 **			*LKOFPODF (−1)*	−0.4752 ***	−0.3935 ***
*LKOFPODJ (−1)*			7.8818 **	8.3822 **			*LKOFPODJ (−1)*	0.9185 ***	1.2828 ***

**Notes:** ***, **, * denotes significance level at 1%, 5% and 10% respectively; prefix D denotes Differences; prefix L denotes Logarithm; Dum denotes dummy; C denotes constant; Values in bold and italic are the ECM.

## Data Availability

Publicly available datasets were analysed in this study. This data can be found here: https://www.eia.gov/environment/data.php (accessed on 12 November 2020); https://unctadstat.unctad.org/wds/ReportFolders/reportFolders.aspx (accessed on 12 November 2020); https://data.worldbank.org/indicator/NY.GDP.MKTP.KD (accessed on 13 November 2020); https://databank.worldbank.org/source/worldwide-governance-indicators (accessed on 13 November 2020); https://www.eia.gov/totalenergy/data/annu (accessed on 12 November 2020); https://kof.ethz.ch/en/forecasts-and-indicators/indicators/kof-globalisation-index.html (accessed on 13 November 2020).

## Appendix A

**Table A1:** Hausman test and F-test for CO_2_ model (2000–2017).

	Developed Countries		Developing Countries	
	I-CO_2_ Model with FDI_si	II-CO_2_ Model with FDI_so	V-CO_2_ Model with KOFSODF	VI-CO_2_ Model with KOFSODJ
F-test	4.85 ***	4.94 ***	3.14 ***	3.19 ***
Hausman test	62.73 ***	63.60 ***	36.03 ***	36.45 ***

**Notes:** *** denotes significance level at 1%.

**Table A2:** Specification tests for CO_2_ model (2000–2017).

	Developed Countries		Developing Countries	
	I-CO_2_ Model with FDI_si	II-CO_2_ Model with FDI_so	V-CO_2_ Model with KOFSODF	VI-CO_2_ Model with KOFSODJ
Pesaran test	7.509 ***	7.788 ***	2.544 **	2.209 **
Frees test	0.332 ***	0.461 ***	0.120	0.035
Fiedman test	51.188 ***	51.497 ***	28.980 ***	27.966 ***
Modified Wald test	297.30 ***	281.42 ***	184.68 ***	215.26 ***
Wooldrige test	125.361 ***	98.212 ***	45.098 ***	52.334 ***

**Notes:** ***, ** denotes significance level at 1%, and 5%, respectively.

**Table A3:** Estimations of ARDL model—both models CO_2_ and ISEW from 2000 to 2017.

	Developed Countries				Developing Countries				
	CO_2_ Model		ISEW Model		ISEW Model			CO_2_ Model	
	I-FDI_si	II-FDI_so	III-FDI_si	IV-FDI_so	VII-KOFSODF	VIII-KOFSODJ		V-KOFSODF	VI-KOFSODJ
*DLGDP_pc*	0.5847***	0.6435 ***					*DLGDP_pc*		
*DLCO2_pc*							*DLCO2*		
*DFDI_fi*	−0.0009 ***	−0.0009 ***			0.015 ***	0.0128 ***	*DFDI_fi*		
*DFDI_fo*							*DFDI_fo*	−0.0112 **	−0.0115 **
*DFDI_si*							*DFDI_si*	0.0024 **	0.0018 *
*DFDI_so*							*DFDI_so*		
*DLFF_p*	0.905 ***	0.9220 ***					*DLFF_p*	1.8024 ***	1.7209 ***
*DLRES_p*							*DLRES_p*		−0.0245 **
*DCORR*			0.0976 **	0.0892 **	0.2731 **		*DCORR*		
*DRQ*			0.0693 **		−0.2738 *		*DRQ*		
*DLKOFECDF*			−0.8967 ***	−0.9049 ***			*DLKOFECDF*		
*DLKOFECDJ*						−0.5456 ***	*DLKOFECDJ*	0.1956 ***	0.1809 ***
*DLKOFSODF*			0.9839 ***	0.837 **	0.4057 **		*DLKOFSODF*		
*DLKOFSODJ*						0.9309 ***	*DLKOFSODJ*		
*DLKOFPODF*							*DLKOFPODF*		
*DLKOFPODJ*							*DLKOFPODJ*		
*LISEW_pc (­−1)*			***−0.1407 ******	***−0.1401 ******	***−0.7447 ******	***−0.7586 ******	*LISEW_pc (−1)*		
*LCO2_pc (−1)*	***−0.3448 ******	***−0.3264 ******	0.2863 ***	0.2597 ***	0.3267 **	0.2485 **	*LCO2 (−1)*	***−0.2775 ******	***−0.2946 ******
*LGDP_pc (−1)*							*LGDP_pc (−1)*		
*FDI_fi (−1)*							*FDI_fi (−1)*	−0.0046 **	−0.0034 **
*FDI_fo (−1)*							*FDI_fo (−1)*		
*FDI_si (−1)*	−0.0002 *		0.0012 ***		0.008 ***	0.0051 ***	*FDI_si (−1)*	0.0012 **	
*FDI_so (­­−1)*		−0.0002 **		0.00068 ***			*FDI_so (−1)*		
*LFF_p (−1)*	0.5075 ***	0.4883 ***					*LFF_p (−1)*	0.6997 ***	0.7817 ***
*LRES_p (−1)*			−0.0496 ***	−0.0658 ***			*LRES_p (−1)*		
*CORR (−1)*			0.1028 ***	0.0895 ***	0.3039 **	0.2419 **	*CORR (−1)*		
*RQ (−1)*							*RQ (−1)*		
*LKOFECDF (−1)*							*LKOFECDF (−1)*		
*LKOFECDJ (−1)*			−0.5117 ***	−0.5396 ***	−0.4261 *	−0.5397 **	*LKOFECDJ (−1)*		
*LKOFSODF (−1)*	−0.2894 ***	−0.2357 ***			0.7076 ***		*LKOFSODF (−1)*	0.0755 **	
*LKOFSODJ (−1)*						1.5626 ***	*LKOFSODJ (−1)*		0.1910 **
*LKOFPODF (−1)*	−0.3895 ***		−0.8895 ***	−0.8307 ***			*LKOFPODF (−1)*	−0.1576 ***	−0.1181 **
*LKOFPODJ (−1)*	0.2196 **		1.1089 ***	1.1739 ***			*LKOFPODJ (−1)*	0.4386 **	0.4674 **
*C*	−4.0770 ***	−4.7775 ***	5.8755 ***	5.2215 ***	8.5173 ***	4.4613 *	*C*	−3.8549 **	−4.8995 ***
*Dum_2009*			−0.1923 ***	−0.2006 ***			*Dum_2009*	−0.1007 ***	−0.0979 ***
*Dum_2010*					0.0806 **	0.14405 ***	*Dum_2010*		
*Dum_2014*	−0.0212 ***	−0.0210 ***							
**Elasticities**									
*LCO2_pc (−1)*			2.0346 *	1.8545 *	0.4387 ***	0.3276 **	*LCO2 (−1)*		
*LGDP_pc (−1)*							*LGDP_pc (−1)*		
*FDI_fi (−1)*							*FDI_fi (−1)*	−0.0166 ***	−0.0117 ***
*FDI_fo (−1)*							*FDI_fo (−1)*		
*FDI_si (−1)*	−0.0004 **		0.0088 **		0.0107 ***	0.0067 ***	*FDI_si (−1)*	0.0045 ***	
*FDI_so (−1)*		−0.0006 ***		0.0049 **			*FDI_so (−1)*		
*LFF_p (−1)*	1.4719 ***	1.4957 ***					*LFF_p (−1)*	2.5214 ***	2.654 ***
*LRES_p (−1)*			−0.3522 ***	−0.4698 ***			*LRES_p (−1)*		
*CORR (−1)*			0.7308 ***	0.6391 **	0.4081 **	0.3189 **	*CORR (−1)*		
*RQ (−1)*							*RQ (−1)*		
*LKOFECDF (−1)*							*LKOFECDF (−1)*		
*LKOFECDJ (−1)*			−3.6371 *	−3.8532 *	−0.5721 **	−0.7115 ***	*LKOFECDJ (−1)*		
*LKOFSODF (−1)*	−0.8394 ***	−0.7221 ***			0.9501 ***		*LKOFSODF (−1)*	0.272 **	
*LKOFSODJ (−1)*						2.06 ***	*LKOFSODJ (−1)*		0.6484 **
*LKOFPODF (−1)*	-1.1295 ***		−6.3223 **	−5.9316 **			*LKOFPODF (−1)*	−0.5677 ***	−0.4009 ***
*LKOFPODJ (−1)*	0.6370***		7.8818 **	8.3822 **			*LKOFPODJ (−1)*	1.5803 ***	1.5869 ***

**Notes:** ***, **, * denotes significance level at 1%, 5% and 10%, respectively; prefix D denotes Differences; prefix L denotes Logarithm; Dum denotes dummy; C denotes constant; Values in bold and italic are the ECM.
